# Inside the November 2023 Issue

**DOI:** 10.24908/pocus.v8i2.16970

**Published:** 2023-11-27

**Authors:** Benjamin T. Galen

**Affiliations:** 1 Department of Medicine, Albert Einstein College of Medicine Bronx, NY USA

**Keywords:** POCUS Journal, Commentary, Letter from the editor

Dear Readers, 

This is a very exciting time for POCUS Journal. As the world’s leading point of care ultrasound journal, we remain free for both authors and readers. Our content brings the POCUS community together as we strive to showcase POCUS use by clinicians from a wide variety of fields in every possible clinical setting. 

The era of being indexed on PubMed has attracted many high-quality submissions, from truly novel case reports to late breaking, practice-changing research on POCUS. The November issue houses such great scholarship from the around the world it is difficult to select the highlights. Tierney et al. (pages 185-192) studied a prospective cohort of hospitalized patients and found that the availability and use of POCUS could reduce hospitalization cost, radiology cost, and chest x-rays. This is a major finding that provides concrete evidence for what POCUS users have believed for many years, but now finally have the proof to garner continued support for their POCUS programs. Saati, et al. (pages 159-164) conducted a pilot study showing that patients can perform their own POCUS exams after brief teleguidance training. This proof-of-concept has broad implications for the POCUS field as we evolve to use new technologies to find better ways to provide individualized care to patients in the future. 

**Figure 1:**
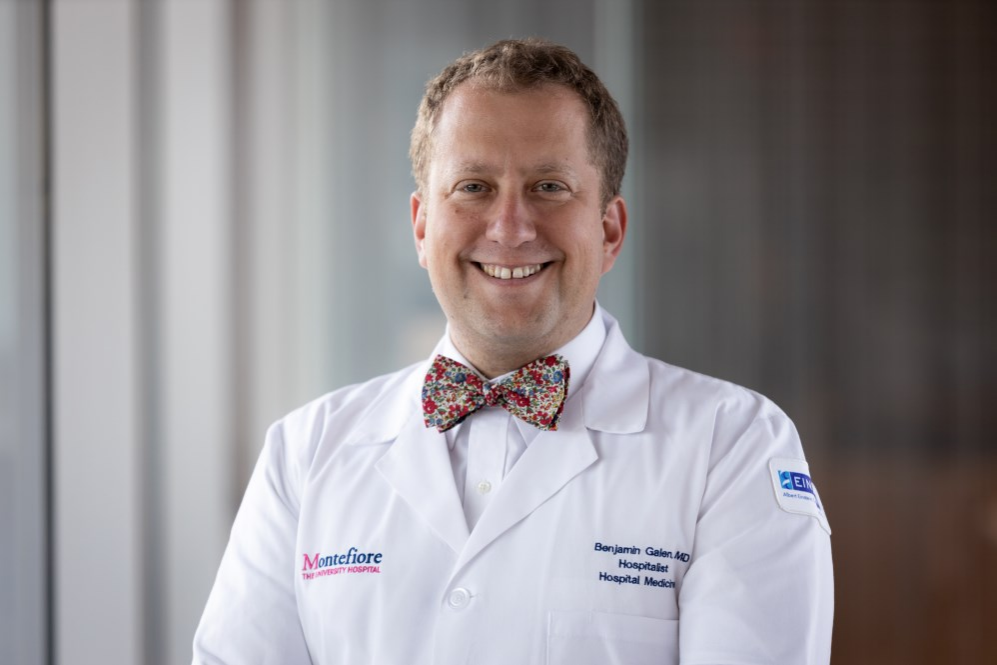
Dr. Benjamin T. Galen, Editor-in-Chief, POCUS Journal

Alas, the November issue of POCUS Journal brings with it some bittersweet news. It will be the last issue for our founding managing editor Julia Herr, MSc, to whom all of us in the POCUS community owe a debt of gratitude. Her dedication to this journal and pursuit of excellence in publishing have brought the POCUS Journal platform to where it is today. 

Please find our author guidelines here: https://pocusjournal.com/author-guidelines/

Sincerely,

Benjamin T. Galen, MD

Department of Medicine, Albert Einstein College of Medicine, Bronx, NY

Editor-in-Chief, POCUS Journal

